# Sensitive proportion in ranked set sampling

**DOI:** 10.1371/journal.pone.0256699

**Published:** 2021-08-31

**Authors:** Azhar Mehmood Abbasi, Muhammad Yousaf Shad

**Affiliations:** Department of Statistics, Quaid-i-Azam University, Islamabad, Pakistan; Roswell Park Cancer Institute, UNITED STATES

## Abstract

This paper considers the concomitant-based rank set sampling (CRSS) for estimation of the sensitive proportion. It is shown that CRSS procedure provides an unbiased estimator of the population sensitive proportion, and it is always more precise than corresponding sample sensitive proportion (Warner SL (1965)) that based on simple random sampling (SRS) without increasing sampling cost. Additionally, a new estimator based on ratio method is introduced using CRSS protocol, preserving the respondent’s confidentiality through a randomizing device. The numerical results of these estimators are obtained by using numerical integration technique. An application to real data is also given to support the methods.

## 1 Introduction

In some social surveys, we may encounter the problem of estimating the proportion of the population having sensitive attribute, such as drug addicts, users of heron and non-taxpayers, for which people are not inclined to respond truthfully. In such situations the techniques for collecting direct information may result in elusive, ambiguous, and even no response. To overcome these problems, [1] advised randomized response (RR) technique under simple random sampling (SRS) plan with the objective to collect truthful answers while fully preserving the respondent’s privacy. This method involves a randomizing device, such as a spinning arrow or a deck of cards, to procure truthful information on the sensitive attribute. The respondent answers ‘yes’ or ‘no’ according to the outcome produced by the randomizing device. As the interviewer is kept unaware of the result produced by the said device, the use of this technique ensures that the respondent cannot be recognized on the basis of his/her response. After development of the first randomized response model [1], numerous variants have been suggested by different researchers to obtain more reliable estimates of the sensitive attribute by increasing respondent’s degree of privacy. A comprehensive literature will not be demonstrated. However, some worth-mentioning work developed under SRS plan can be found in the Reference [2–5] and the references cited therein.

Ranked set sampling (RSS) was introduced by [6], as an efficient alternative to simple random sampling (SRS), for estimation of pasture and forage yields. The RSS employs ranking of the small sets of units by visually or via a concomitant information before selecting final sample for actual quantification. [7] developed the theory of RSS procedure. More detail and application of RSS (CRSS) can be explored in the References [8–13].

The estimation of non-sensitive population proportion under CRSS has been investigated by [14]. Thereafter, [15] introduced a new proportion estimator in CRSS framework and showed that it works better than that of [14] without using extra resources. Recently, [16] has highlighted some drawbacks associated with the estimator given in [15] and proposed new improved estimators. Moreover, [17] showed that how RSS can be applied to ordered categorical variables for estimating the probabilities of all categories. They used ordinal logistic regression to aid in the ranking of the ordinal variable of interest.

The idea of using RSS in the estimation of sensitive attribute is similar to its application in above discussed inference problems. The ranking can be carried out by visually or by using a concomitant variable which should be non sensitive but statistically correlated with study attribute. The following two examples develop better understanding about how to use concomitant information for ranking the units:

**Example 1.** Let us suppose that we want to estimate the proportion of drug addicts in a social survey through RR technique. We can easily rank (order) two or more units by a glance with respect to either their facial expressions or ages.
**Example 2.** Let us suppose that under study parameter is the proportion of non-taxpayers. We can order two or more households by a glance with respect to either their living styles or house-sizes.

Recently, [18] has adopted model-based ranking approach, introduced by [17], for studying sensitive proportion using concomitant based-rank set sampling. This ranking method requires estimated success probabilities by fitting the logistic regression. The main concern with this ranking method is that concomitant information is not directly used for ranking of the study variable, instead it requires fitting logistic model to the data on previous studies and ranking process is done on the basis of obtained probabilities. In this paper, a new efficient estimator is proposed using CRSS which overcomes the drawbacks of [18] procedure and also beats [1] estimator. Furthermore, a new estimator based on ratio method is also introduced under CRSS plan.

## 2 Background

Let (*Y*, *X*) denotes a bivariate random variable where sensitive study attribute *Y* follows a Bernoulli distribution and *X* is a continuous nonsensitive concomitant with cumulative distribution function (cdf) *F*_*X*_(*x*). Suppose that the conditional distribution of *Y* given *X* = *x* is also Bernoulli and is denoted by *B*(1, *g*(*x*)), where *g*(*x*) ∈ (0, 1) could be inverse logit (probit) link function defined as

$$\begin{array}{c} g\left(x\right)=\{\begin{array}{c} \frac{\text{exp}(\beta_{0}+\beta_{1}x)}{1+\text{exp}(\beta_{0}+\beta_{1}x)}\;\text{logit}\;\text{function}\\ \frac{1}{\sqrt{2\pi }}\int_{-\infty }^{w}e^{-\frac{1}{2}x^{2}}\;\text{dx}\;\text{probit}\;\text{function,} \end{array} \end{array}$$

where *w* = 0.551(*β*_0_ + *β*_1_ *x*), *α*, *β* ∈ ℜ.

It follows that the marginal distribution of *Y* is B(1, *π*) with mean *π* = E[*g*(*X*)] and variance $\sigma_{y}^{2}=\pi \left(1-\pi \right)$. For more detailed discussion, an interested reader can pursue [14]. The CRSS plan for selection of *m*(≥ 2) units can be elucidated as:

**Step 1** Identify *m*^2^ units from (*Y*, *X*) and divide them into *m* sets of size *m*.
**Step 2** From each set of size *m*, obtain the exact measurement on *X*, then rank the sets according to the values of *X*.
**Step 3** Obtain the corresponding *Y* values of the *i*th (*i* = 1, 2, …, *m*) ordered unit of *X* in the *i*th set.
**Step 4** The above Steps 1–3 can be repeated for *n* cycles, if required, to obtain a sample of size *k* = *mn*.

Let {(*Y*_[1]*j*_, *X*_(1)*j*_), (*Y*_[2]*j*_, *X*_(2)*j*_), …, (*Y*_[*i*]*j*_, *X*_(*i*)*j*_), …, (*Y*_[*m*]*j*_, *X*_(*m*)*j*_)} be a bivariate ranked set sample of size *m* in *j*th cycle, *j* = 1, 2, …, *n*, where *Y*_[*i*]*j*_ denotes *i*th *imperfect* ranked unit in the *j*th cycle and *X*_(*i*)*j*_ denotes *i*th *perfect* ranked unit in *j*th cycle. Note that the square bracket [⋅] denotes imperfect ranking and (⋅) serves for perfect ranking. Again, from [14], *Y*_[*i*]_ is *B*(1, *π*_[*i*]_) with mean (probability) *π*_[*i*]_ = E[*g*(*X*_(*i*)_)] and variance $\sigma_{y_{\left[i\right]}}^{2}=\pi_{\left[i\right]}\left(1-\pi_{\left[i\right]}\right)$. Let *f*_(*i*)_(*x*) be the probability density function (pdf) and *F*_(*i*)_(*x*) cumulative distribution function (cdf) of an order statistics (OS) *X*_(*i*)_ then we have

f(i)(x)=(mi)(F(x))i-1(1-F(x))m-if(x),-∞<x<∞. (2.1)

and

F(i)(x)=∑r=im(nr)(F(x))r(1-F(x))m-r

The mean and variance of *X*_(*i*)_ are given by

$$\begin{array}{c} {\bar{X}}_{\left(i\right)}=\int_{-\infty }^{\infty }xf_{\left(i\right)}\left(x\right)\text{dx}\;\text{and}\;\sigma_{x(i)}^{2}=\int_{-\infty }^{\infty }{\left(x-\mu_{x(i)}\right)}^{2}f_{\left(i\right)}\left(x\right)\text{dx} \end{array}$$

respectively; see the Reference [19].

The success (‘yes’) probability of *Y*_[*i*]_, as given in [14], is numerically computed as

$$\begin{array}{c} \text{E}\left(Y_{\left[i\right]}\right)=\pi_{\left[i\right]}=\int_{-\infty }^{\infty }f_{\left(i\right)}\left(x\right)g\left(x\right)\text{dx} \end{array}$$

The covariance between *X*_(*i*)_ and *Y*_[*i*]_ is defined as

$$\begin{array}{c} \text{Cov}\left(X_{\left(i\right)},Y_{\left[i\right]}\right)=\text{E}\left(X_{\left(i\right)}Y_{\left[i\right]}\right)-\text{E}\left(X_{\left(i\right)}\right)\text{E}\left(Y_{\left[i\right]}\right), \end{array}$$

By virtue of partitioning, as defined in [20], we have

$$\begin{array}{cc} f(x)=\frac{1}{m}\sum_{i=1}^{m}f_{\left(i\right)}\left(x\right) & \;f(y)=\frac{1}{m}\sum_{i=1}^{m}f_{\left[i\right]}\left(y\right) \end{array}$$ (2.2)

The following well-known notations will be used in this study.

$$\begin{array}{cccccccc} \pi & =\text{E}(Y) & \;\sigma_{y}^{2} & =\text{E}{\left(Y-\pi \right)}^{2} & \;d_{x(i)} & ={\bar{X}}_{\left(i\right)}-\bar{X}\\ \pi_{\left[i\right]} & =\text{E}(Y_{\left[i\right]}) & \;\sigma_{y_{\left[i\right]}}^{2} & =\text{E}{\left(Y_{\left[i\right]}-\pi_{\left[i\right]}\right)}^{2} & \;d_{y[i]} & =\pi_{\left[i\right]}-\pi \\ \bar{X} & =\text{E}(X) & \;\sigma_{x}^{2} & =\text{E}{\left(X-\bar{X}\right)}^{2} & \;d_{xy_{\left[i\right]}} & =\left({\bar{X}}_{\left(i\right)}-\bar{X}\right)\left(\pi_{\left[i\right]}-\pi \right) & & \\ {\bar{X}}_{\left(i\right)} & =\text{E}(X_{\left(i\right)}) & \;\sigma_{x_{\left(i\right)}}^{2} & =\text{E}{\left(X_{\left(i\right)}-{\bar{X}}_{\left(i\right)}\right)}^{2} & \;\sigma_{xy_{\left[i\right]}} & =\text{E}\left(X_{\left(i\right)}-{\bar{X}}_{\left(i\right)}\right)\left(Y_{\left[i\right]}-\pi_{\left[i\right]}\right) \end{array}$$

Also the following relationships will be used in this paper.

$$\begin{array}{cccccc} \sum_{i=1}^{m}\pi_{\left[i\right]} & =m\pi & \sum_{i=1}^{m}d_{x(i)} & =0 & \;\;\sum_{i=1}^{m}\sigma_{y_{\left[i\right]}}^{2} & =m\sigma_{y}^{2}-\sum_{i=1}^{m}d_{y[i]}^{2}\\ \sum_{i=1}^{m}{\bar{X}}_{\left(i\right)} & =m\bar{X} & \;\sum_{i=1}^{m}d_{y_{\left[i\right]}} & =0 & \;\sum_{i=1}^{m}\sigma_{x_{\left(i\right)}}^{2} & =m\sigma_{x}^{2}-\sum_{i=1}^{m}d_{x(i)}^{2}\\ \sum_{i=1}^{m}\sigma_{xy_{\left[i\right]}} & =m\sigma_{xy}-\sum_{i=1}^{m}d_{xy_{\left[i\right]}} \end{array}$$

For more detail, see the References [20, 21].

## 3 Warner’s model under CRSS

As this study involves randomized response (RR) procedure, it is important to give an overview of the basic RR procedure given in the Reference [1]. Let *Y*_1*j*_, *Y*_2*j*_, …, *Y*_*mj*_ be a simple random sample with replacement (SRSWR) of size *m* in *j*th cycle, for (*j* = 1, 2, …, *n*). Each respondent is provided with a suitable randomizing device, say a spinner, for selection of one of the two statements: (a) *I have the sensitive attribute* A (b) *I do not have the sensitive attribute* A with pre-assigned selection probabilities *p* ≠ 0.5 and 1 − *p* respectively. Each respondent spins the spinner and report ‘yes’ (‘no’) if his/her status matches (does not match) with the statement pointed out by the randomization device. As the interviewer is kept unaware of the outcome of the randomization device, and this makes the respondent comfortable to truthfully report his/her actual status. Then ‘yes’ response of *i*th (*i* = 1, 2, …, *m*) respondent at *j*th cycle is given by

$$\begin{array}{c} \lambda =P(yes)=p\pi +(1-p)(1-\pi ) \end{array}$$

Let *m*_1_ denotes number of ‘yes’ responses out of the sample of size *k* = *mn*, then [1] derived the maximum likelihood estimate of *π* as given by ${\hat{\pi }}_{\left(srs\right)}=\left\{\hat{\lambda }-\left(1-p\right)\right\}/\left(2p-1\right)$ where $\hat{\lambda }=m_{1}/k$ is estimate of λ. The estimator ${\hat{\pi }}_{\left(srs\right)}$ is unbiased and its variance is given by

$$\begin{array}{c} \text{Var}\left({\hat{\pi }}_{\left(srs\right)}\right)=\frac{1}{n}\{\frac{\sigma_{y}^{2}}{m}+\frac{p(1-p)}{m{\left(2p-1\right)}^{2}}\}=\frac{\sigma_{y}^{2}}{k}+\frac{p(1-p)}{k{\left(2p-1\right)}^{2}} \end{array}$$ (3.1)

Now, suppose that the respondents are selected using CRSS design and are instructed to choose one of the two above-mentioned statements (a) and (b) by using the given randomizing device. The respondent reports ‘yes’ (‘no’) according to the outcomes of the randomizing device and his/her actual status. A complete layout of *i*th response under CRSS is given in the S1 Fig. Let *Y*_[*i*]*j*_ = 1 if *i*th ranked unit reports ‘yes’, otherwise *Y*_[*i*]*j*_ = 0. Then

$$\begin{array}{cc} P(Y_{[i]j}=1) & =p\pi_{\left[i\right]}+\left(1-p\right)\left(1-\pi_{\left[i\right]}\right)\\ P(Y_{[i]j}=0) & =p\left(1-\pi_{\left[i\right]}\right)+\left(1-p\right)\pi_{\left[i\right]}. \end{array}$$

Let *Y*_[*i*]1_, *Y*_[*i*]2_, …, *Y*_[*i*]*n*_ are independent and identically distributed (i.i.d) Bernoulli randomized responses under CRSS plan with parameter *pπ*_[*i*]_ + (1 − *p*)(1 − *π*_[*i*]_), then likelihood function of *π*_[*i*]_ for the given data *Y*_[*i*]*j*_, *j* = 1, 2, … *n* is

$$\begin{array}{cc} L(\pi_{\left[i\right]}|y_{\left[i\right]}) & =\prod_{j=1}^{n}{\left[p\pi_{\left[i\right]}+\left(1-p\right)\left(1-\pi_{\left[i\right]}\right)\right]}^{y_{[i]j}}{\left[p\left(1-\pi_{\left[i\right]}\right)+\left(1-p\right)\pi_{\left[i\right]}\right]}^{1-y_{[i]j}}\\ & ={\left[p\pi_{\left[i\right]}+\left(1-p\right)\left(1-\pi_{\left[i\right]}\right)\right]}^{z_{i}}{\left[p\left(1-\pi_{\left[i\right]}\right)+\left(1-p\right)\pi_{\left[i\right]}\right]}^{n-z_{i}},i=1,2,\ldots ,m. \end{array}$$ (3.2)

where $z_{i}=\sum_{j=1}^{n}y_{[i]j}$ is the total number of successes observed under *i*th ranking unit. It is obvious that *Z*_*i*_ is binomial variate with parameters *n* and *pπ*_[*i*]_ + (1 − *p*)(1 − *π*_[*i*]_). The joint likelihood function of *π*_[*i*]_, *i* = 1, 2, …, *m* given CRSS data *y*_crss_ = {*Y*_[*i*]*j*_, *i* = 1, 2, …, *m*;*j* = 1, 2, …, *n*} is

$$\begin{array}{c} \text{L}(\pi |y_{\text{[crss]}})=\prod_{i=1}^{m}L\left(\pi_{\left[i\right]}|Y_{\left[i\right]}\right)=\prod_{i=1}^{m}{\left[p\pi_{\left[i\right]}+\left(1-p\right)\left(1-\pi_{\left[i\right]}\right)\right]}^{z_{i}}{\left[p\left(1-\pi_{\left[i\right]}\right)+\left(1-p\right)\pi_{\left[i\right]}\right]}^{n-z_{i}} \end{array}$$ (3.3)

Note that the form of maximum likelihood (ML) function given in (3.3) is too complicated to obtain ML estimate of *π*. Moreover, the variance of the estimator from (3.3) will not in closed form. To avoid this situation, we separately estimate each *π*_[*i*]_ using likelihood function given in (3.2) and then these individual proportions are combined by using the relation $\pi =\frac{1}{m}\sum_{i=1}^{m}\pi_{\left[i\right]}$ for overall estimate of *π*. The log of the likelihood function (3.2) is

$$\begin{array}{cc} \text{logL}(\pi_{\left[i\right]}|y_{\left[i\right]}) & =z_{i}\text{log}\left[p\pi_{\left[i\right]}+\left(1-p\right)\left(1-\pi_{\left[i\right]}\right)\right]+\left(n-z_{i}\right)\text{log}\left[p\left(1-\pi_{\left[i\right]}\right)+\left(1-p\right)\pi_{\left[i\right]}\right].\\ & =z_{i}\text{log}\left[\left(2p-1\right)\pi_{\left[i\right]}+\left(1-p\right)\right]+\left(n-z_{i}\right)\text{log}\left[-\left(2p-1\right)\pi_{\left[i\right]}+p\right)]. \end{array}$$

and necessary conditions on *π*_[*i*]_ for a maximum give

$$\begin{array}{cc} \frac{z_{i}\left(2p-1\right)}{\left(2p-1\right)\pi_{\left[i\right]}+\left(1-p\right)} & =\frac{\left(n-z_{i}\right)\left(2p-1\right)}{-\left(2p-1\right)\pi_{\left[i\right]}+p} \end{array}$$

After simplification, we obtain

$$\begin{array}{cc} {\hat{\pi }}_{\left[i\right]} & =\frac{{\hat{\lambda }}_{\left[i\right]}-\left(1-p\right)}{2p-1},\;i=1,2,\ldots ,m. \end{array}$$

where ${\hat{\lambda }}_{\left[i\right]}=z_{i}/n$. Hence, the propose measure of *π* under CRSS plan is given by

$$\begin{array}{c} {\hat{\pi }}_{\left(crss\right)}=\frac{1}{m}\sum_{i=1}^{m}{\hat{\pi }}_{\left[i\right]}=\frac{{\hat{\lambda }}_{\left[crss\right]}-\left(1-p\right)}{2p-1}, \end{array}$$ (3.4)

${\hat{\lambda }}_{\left[crss\right]}=\frac{1}{m}\sum_{i=1}^{m}{\hat{\lambda }}_{\left[i\right]}$.

**Theorem**: Let {*Y*_[*i*]*j*_, *i* = 1, 2, …, *m*;*j* = 1, 2, …, *n*} be a ranked set sample of size *k*. Then

**(i)**${\hat{\pi }}_{\left(crss\right)}$ is an unbiased estimator of the population proportion *π* i.e., E(${\hat{\pi }}_{\left(crss\right)}$) = *π*
**(ii)**${\hat{\pi }}_{\left(crss\right)}$ is more precise than ${\hat{\pi }}_{\left(srs\right)}$ i.e., $\text{Var}\left({\hat{\pi }}_{\left(crss\right)}\right)=\text{Var}\left({\hat{\pi }}_{\left(srs\right)}\right)-\frac{1}{km}\sum_{i=1}^{m}d_{y_{\left[i\right]}}^{2}\leq \text{Var}\left({\hat{\pi }}_{\left(srs\right)}\right)$

**Proof**:

(i) From (3.4), we have
  $$\begin{array}{cc} \text{E}({\hat{\pi }}_{\left(crss\right)}) & =\frac{\text{E}({\hat{\lambda }}_{\left[crss\right]})-\left(1-p\right)}{2p-1} \end{array}$$
  But
  $$\begin{array}{cc} \text{E}({\hat{\lambda }}_{\left[crss\right]}) & =\frac{1}{m}\sum_{i=1}^{m}\text{E}\left({\hat{\lambda }}_{\left[i\right]}\right)\\ & =\frac{1}{m}\sum_{i=1}^{m}\left[p\pi_{\left[i\right]}+\left(1-p\right)\left(1-\pi_{\left[i\right]}\right)\right]\\ & =p\pi +(1-p)(1-\pi )\\ & =(2p-1)\pi +(1-p)\\ & =\lambda \end{array}$$
  Hence
  $$\begin{array}{cc} \text{E}({\hat{\pi }}_{\left(crss\right)}) & =\frac{\lambda -(1-p)}{2p-1}\\ & =\frac{\pi (2p-1)+(1-p)-(1-p)}{2p-1}\\ & =\pi \end{array}$$
  This completes proof (i).
(ii) From (3.4), the variance of ${\hat{\pi }}_{\left(crss\right)}$ is
  $$\begin{array}{c} \text{Var}({\hat{\pi }}_{\left(crss\right)})=\text{Var}(\frac{{\hat{\lambda }}_{\left[crss\right]}-\left(1-p\right)}{2p-1}) \end{array}$$ (3.5)

Since variance of a constant term is zero, (3.5) reduces to

$$\begin{array}{cc} \text{Var}({\hat{\pi }}_{\left(crss\right)}) & =\frac{1}{{\left(2p-1\right)}^{2}}\text{Var}\left({\hat{\lambda }}_{\left[crss\right]}\right)\\ & =\frac{1}{{\left(2p-1\right)}^{2}}\frac{1}{m^{2}}\sum_{i=1}^{m}\text{Var}\left({\hat{\lambda }}_{\left[i\right]}\right)\\ & =\frac{1}{{\left(2p-1\right)}^{2}}\frac{1}{km}\sum_{i=1}^{m}\lambda_{\left[i\right]}\left(1-\lambda_{\left[i\right]}\right), \end{array}$$ (3.6)

where λ_[*i*]_ = *pπ*_[*i*]_ + (1 − *p*)(1 − *π*_[*i*]_) = (2*p* − 1)*π*_[*i*]_ + (1 − *p*).

Now, substituting the value of λ_[*i*]_ in (3.6) and then simplification gives

$$\begin{array}{cc} \text{Var}({\hat{\pi }}_{\left(crss\right)}) & =\frac{1}{km}\sum_{i=1}^{m}\sigma_{y_{\left[i\right]}}^{2}+\frac{p(1-p)}{k{\left(2p-1\right)}^{2}}.\\ & =\frac{1}{km}\left(m\sigma_{y}^{2}-\sum_{i=1}^{m}d_{y[i]}^{2}\right)+\frac{p(1-p)}{k{\left(2p-1\right)}^{2}}\\ & =\frac{1}{k}\sigma_{y}^{2}+\frac{p(1-p)}{k{\left(2p-1\right)}^{2}}-\frac{1}{km}\sum_{i=1}^{m}d_{y[i]}^{2}\\ & =\text{Var}\left({\hat{\pi }}_{\left(srs\right)}\right)-\frac{1}{km}\sum_{i=1}^{m}d_{y_{\left[i\right]}}^{2} \end{array}$$

We have used the fact $\sum_{i=1}^{m}\sigma_{y_{\left[i\right]}}^{2}=m\sigma_{y}^{2}-\sum_{i=1}^{m}d_{y[i]}^{2}.$ Note that $\frac{1}{km}\sum_{i=1}^{m}d_{y_{\left[i\right]}}^{2}\geq 0$, hence $\text{Var}\left({\hat{\pi }}_{\left(crss\right)}\right)\leq \text{Var}\left({\hat{\pi }}_{\left(srs\right)}\right)$. This completes the proof (ii).

The relative efficiency (RE) of ${\hat{\pi }}_{\left(crss\right)}$ with respect to ${\hat{\pi }}_{\left(srs\right)}$ can be examined by the ratio

$$\begin{array}{c} \text{RE}\left[{\hat{\pi }}_{\left(crss\right)},{\hat{\pi }}_{\left(srs\right)}\right]=\frac{\text{Var}({\hat{\pi }}_{\left(srs\right)})}{\text{Var}({\hat{\pi }}_{\left(crss\right)})}={\left\{1-\frac{\sum_{i=1}^{m}d_{y_{\left[i\right]}}^{2}}{m^{2}\text{Var}\left({\hat{\pi }}_{\left(srs\right)}\right)}\right\}}^{-1} \end{array}$$ (3.7)

The expression (3.7) is always greater than unity irrespective of the choice of *g*(⋅), subject to the condition that *p* ≠ 0.5. In other words, ${\hat{\pi }}_{\left(crss\right)}$ is a superior alternative to ${\hat{\pi }}_{\left(srs\right)}$. It may be noted that when *p* = 0.5, $\text{Var}({\hat{\pi }}_{srs})\to \infty$ and consequently $\text{RE}[{\hat{\pi }}_{\left(crss\right)},{\hat{\pi }}_{\left(srs\right)}]=1$. It is also obvious from (3.7) that RE is independent of number of cycles *n*, i.e., the results can not be improved by increasing *n*. The following result also holds from the Reference [14] when *m* is fixed and *n* → ∞.

$$\begin{array}{c} \sqrt{nm}\left({\hat{\pi }}_{\left(crss\right)}-\pi \right)\to \text{Normal}(0,\frac{1}{m}\sum_{i=1}^{m}\sigma_{y_{\left[i\right]}}^{2}+\frac{p(1-p)}{{\left(2p-1\right)}^{2}}). \end{array}$$ (3.8)

We can see that when *m* = 1, (3.8) simplifies to Warner’s result [1] under SRS. Furthermore, the choice *p* = 1 i.e., selection of sensitive attribute by randomization device is sure and respondent’s privacy is zero, (3.8) reduces to [14] procedure of directly asking the respondent about the attribute of interest under CRSS. Whereas the choice *p* = 0 i.e., no chance of selecting sensitive question by randomizing device, also brings (3.8) to [14] procedure. Moreover, if both *p* and *m* are equal to 1, (3.8) becomes conventional method of direct interaction with the respondent under SRS. However, for precise and reliable estimate the conditions *m* ≥ 2, 0 < *p* < 0.5(or0.5 < *p* < 1) are required. Finally, a consistent estimator of variance in (3.8) can be obtained by replacing *π*_[*i*]_ with ${\hat{\pi }}_{\left[i\right]}=\sum_{j=1}^{n}Y_{[i]j}/n$. In this way the variance estimate becomes free of *g*(⋅). Hence, asymptotic inference can easily be derived from CRSS plan.

### 3.1 Numerical illustration

We investigate the RE of ${\hat{\pi }}_{\left(crss\right)}$ with respect to ${\hat{\pi }}_{\left(srs\right)}$ by using the expression (3.7) for different choices of *β*_0_, *β*_1_, 0.1 ≤ *p* ≤ 0.9 and assuming *X* follows (i) normal distribution with parameters mean = 2 and variance = 1 (ii) uniform over the range 0 and 1. It is important to recall that the RE formula as given in (3.7) is independent of *n*, hence we take different *m*(= 2, 3, 4, 5) instead of *n* to evaluate the performance of ${\hat{\pi }}_{\left(crss\right)}$. Furthermore, the magnitude of correlation coefficient between *X* and *Y* is also computed under inverse logit (probit) link function. All results are obtained by numerical integration technique, as demonstrated in the Section 2, using Mathematica Software and are displayed in S1-S4 Tables in S1 File.

As expected, the RE is an increasing function of *m* and/(or) *ρ*. It is also symmetric about *p* = 0.5. In other words, for given *m* and *ρ*, it does not matter one assigns the design parameter *p* or 1 − *p* to the aforesaid sensitive statement (a). However, respondents cooperation can be increased by choosing *p* or 1 − *p*, whichever is assigned to the statement (a), from the interval [0.10.5) and at the same time one can also achieve reasonable precision for some suitable choice of *m* and/(or) *ρ*. Generally, the results under both link functions are almost same.

## 4 Sensitive proportion using ratio method

In survey sampling, a concomitant information is commonly used for improving precision of the estimator pertaining to non-sensitive quantity. Such information is utilized at the designing phase for selection of appropriate sample or directly at the estimation phase by ratio (product) or regression methods or incorporated at both phases. As regards sensitive proportion, a few attempts have been made to consider concomitant information at designing or estimation phase under SRS plan. For example, [22] has constructed a ratio estimator for sensitive proportion under SRS plan. The randomizing device for this method consisting of a deck of cards showing two aforesaid statements (a) and (b). In addition, each individual is required to disclose his/her true value of nonsensitive concomitant *X*. Then sensitive proportion estimate under this scenario is estimated as

$$\begin{array}{c} {\hat{\pi }}_{Y(srs)}=\frac{{\hat{\lambda }}_{r}-\left(1-p\right)}{2p-1}, \end{array}$$

where ${\hat{\lambda }}_{r}=\hat{\lambda }\bar{X}/\hat{\bar{X}}$ is ratio estimator of λ. The expressions of bias and MSE of ${\hat{\pi }}_{Y(srs)}$ are, respectively, given by

$$\begin{array}{c} \text{Bias}({\hat{\pi }}_{Y(srs)})=\frac{1}{k}\{\pi \left(C_{x}^{2}-C_{xy}\right)-\frac{1-p}{2p-1}C_{x}^{2}\}, \end{array}$$ (4.1)

and

$$\begin{array}{c} \text{MSE}({\hat{\pi }}_{Y(srs)})=\text{Var}\left({\hat{\pi }}_{srs}\right)-\frac{\lambda }{k{\left(2p-1\right)}^{2}}\{2\left(2p-1\right)\pi C_{xy}-\lambda C_{x}^{2}\}, \end{array}$$ (4.2)

where $C_{xy}=\sigma_{xy}/\left(\bar{X}\pi \right)$ and $C_{x}=\sigma_{x}/\bar{X}$. [22] showed that ${\hat{\pi }}_{Y}$ is more precise than Warner’s estimator [1] under some suitable conditions. Here, it is important to point out that the concomitant variable used in [22] method is binary. However, in case of continuous concomitant variable its functional form will remain the same except estimation process shifted to numerical integration. Thereafter, [23] extended this work and presented a general form of the estimator under SRS plan. To the best of our information, no single attempt has been made so far to consider concomitant information at both designing and estimation stages to optimize gain in precision for estimating sensitive proportion using CRSS plan. This motivated us to fill up this gape in the literature and suggest a new improved procedure.

Let {(*Y*_[*i*]*j*_, *X*_(*i*)*j*_);*i* = 1, 2, …, *m*; *j* = 1, 2, …, *n*} be a bivariate ranked set sample. The respondents are instructed to select one of the two aforesaid statements (a) and (b) by using a randomizing device and report ‘yes’(‘no’) according to the statement selected by the device and their actual status. In addition, each individual is advised to provide his/her true value of *X*. Now, on the lines of [22] estimator, we propose the following estimator under CRSS plan:

$$\begin{array}{c} {\hat{\pi }}_{A(crss)}=\frac{{\hat{\lambda }}_{r,rss}-\left(1-p\right)}{2p-1}, \end{array}$$ (4.3)

where ${\hat{\lambda }}_{r,rss}={\hat{\lambda }}_{\left[rss\right]}\bar{X}/{\hat{\bar{X}}}_{\left(rss\right)}$ is ratio estimator of λ, ${\hat{\lambda }}_{\left[rss\right]}=\frac{1}{k}\sum_{i=1}^{m}\sum_{j=1}^{n}Y_{[i]j}$, ${\hat{\bar{X}}}_{\left(rss\right)}=\frac{1}{k}\sum_{i=1}^{m}\sum_{j=1}^{n}X_{(i)j}$. To derive bias and MSE of the suggested ratio estimator up to the first order of approximation, we proceed as follows:

Let

$$\begin{array}{c} \xi_{o}=\frac{{\hat{\lambda }}_{\left[rss\right]}-\lambda }{\lambda }\;\text{and}\;\xi_{1}=\frac{{\hat{\bar{X}}}_{\left(rss\right)}-\bar{X}}{\bar{X}} \end{array}$$

such that E(*ξ*_0_) = 0 = E(*ξ*_1_). Following [20, 21] and keeping in view randomized response model, we have

$$\begin{array}{c} \text{E}(\xi_{o}^{2})=\frac{1}{k}\{\frac{1}{\lambda^{2}}\left({\left(2p-1\right)}^{2}\pi \left(1-\pi \right)+p\left(1-p\right)\right)-\frac{1}{m}\sum_{i=1}^{m}\tau_{y[i]}^{2}\}\text{E}(\xi_{1}^{2})=\frac{1}{k}\{C_{x}^{2}-\frac{1}{m}\sum_{i=1}^{m}\tau_{x(i)}^{2}\} \end{array}$$

and

$$\begin{array}{c} \text{E}(\xi_{0}\xi_{1})=\frac{1}{k}\{\frac{1}{\lambda }\left(2p-1\right)\pi C_{xy}-\frac{1}{m}\sum_{i=1}^{m}\tau_{xy[i]}\} \end{array}$$

For proof, see S1 Appendix.

Now, expressing (4.3) in terms of *ξ*_*i*_, *i* = 0, 1, we have

$$\begin{array}{cc} {\hat{\pi }}_{A(crss)} & =\frac{1}{2p-1}\{\frac{\lambda \left(1+\xi_{0}\right)\bar{X}}{\bar{X}\left(1+\xi_{1}\right)}-\left(1-p\right)\}\\ & =\frac{1}{2p-1}\{\lambda \left(1+\xi_{0}\right){\left(1+\xi_{1}\right)}^{-1}-\left(1-p\right)\}\\ & =\frac{1}{2p-1}\{\lambda \left(1+\xi_{0}\right)\left(1-\xi_{1}+\xi_{1}^{2}\right)-\left(1-p\right)\}\\ & =\frac{1}{2p-1}\{\lambda \left(1+\xi_{0}-\xi_{1}+\xi_{1}^{2}-\xi_{0}\xi_{1}\right)-\left(1-p\right)\}\\ & =\pi +\frac{\lambda }{2p-1}\{\xi_{0}-\xi_{1}+\xi_{1}^{2}-\xi_{0}\xi_{1}\} \end{array}$$ (4.4)

Applying expectation on both sides of (4.4), we have

$$\begin{array}{cc} \text{E}({\hat{\pi }}_{A(crss)}) & =\pi +\frac{\lambda }{2p-1}\{0+\text{E}\left(\xi_{1}^{2}\right)-\text{E}\left(\xi_{0}\xi_{1}\right)\}\\ & =\pi +\frac{\lambda }{(2p-1)k}\{C_{x}^{2}-\frac{1}{m}\sum_{i=}^{m}\tau_{x(i)}^{2}-\frac{(2p-1)\pi }{\lambda }C_{yx}+\frac{1}{m}\sum_{i=1}^{m}\tau_{yx[i]})\}\\ & =\pi +\frac{\lambda }{(2p-1)k}\{C_{x}^{2}-\frac{(2p-1)\pi }{\lambda }C_{yx}-\frac{1}{m}\sum_{i=}^{m}\tau_{x(i)}^{2}+\frac{1}{m}\sum_{i=1}^{m}\tau_{yx[i]})\} \end{array}$$

So the bias expression in the final form is given by

$$\begin{array}{c} \text{Bias}({\hat{\pi }}_{A(crss)})=\frac{\lambda }{(2p-1)k}\{C_{x}^{2}-\frac{(2p-1)\pi }{\lambda }C_{yx}-\frac{1}{m}\sum_{i=1}^{m}\left(\tau_{x(i)}^{2}-\tau_{yx[i]}\right)\} \end{array}$$ (4.5)

We see that the bias of ${\hat{\pi }}_{A(crss)}$ approaches to zero as *k* becomes infinitely large, indicating ${\hat{\pi }}_{A(crss)}$ is a consistent estimator of *π*. To obtain MSE of ${\hat{\pi }}_{A(crss)}$ up to the first order of approximation, we extract the following expression from (4.4)

$$\begin{array}{c} {\hat{\pi }}_{A(crss)}-\pi \approx \frac{\lambda }{2p-1}\{\xi_{0}-\xi_{1}\} \end{array}$$ (4.6)

By the definition of MSE, from (4.6), we have

$$\begin{array}{cc} \text{MSE}({\hat{\pi }}_{A(crss)}) & =\text{E}{\left({\hat{\pi }}_{A(crss)}-\pi \right)}^{2}\\ & =\frac{\lambda^{2}}{{\left(2p-1\right)}^{2}}\text{E}\{\xi_{0}^{2}+\xi_{1}^{2}-2\xi_{0}\xi_{1}\}\\ & =\frac{\lambda^{2}}{{\left(2p-1\right)}^{2}}\{\text{E}\left(\xi_{0}^{2}\right)+\text{E}\left(\xi_{1}^{2}\right)-2\text{E}\left(\xi_{0}\xi_{1}\right)\}\\ & =\frac{\lambda^{2}}{k{\left(2p-1\right)}^{2}}\{\frac{1}{\lambda^{2}}\left({\left(2p-1\right)}^{2}\pi \left(1-\pi \right)+p\left(1-p\right)\right)-\frac{1}{m}\sum_{i=1}^{m}\tau_{y[i]}^{2}+\\ & \;\;\;C_{x}^{2}-\frac{1}{m}\sum_{i=1}^{m}\tau_{x(i)}^{2}-\frac{2}{\lambda }\left(2p-1\right)\pi C_{xy}+\frac{2}{m}\sum_{i=1}^{m}\tau_{xy[i]}\}\\ & =\{\text{Var}\left({\hat{\pi }}_{srs}\right)-\frac{\lambda }{k{\left(2p-1\right)}^{2}}\{2\left(2p-1\right)\pi C_{xy}-\lambda C_{x}^{2}\}\\ & \;\;\;\;-\frac{\lambda^{2}}{mk{\left(2p-1\right)}^{2}}\sum_{i=1}^{m}(\tau_{y[i]}^{2}+\tau_{x(i)}^{2}-2\tau_{yx[i]})\} \end{array}$$

or

$$\begin{array}{c} \text{MSE}({\hat{\pi }}_{A(crss)})=\text{MSE}\left({\hat{\pi }}_{Y(srs)}\right)-\frac{\lambda^{2}}{mk{\left(2p-1\right)}^{2}}\sum_{i=1}^{m}{\left(\tau_{y[i]}-\tau_{x(i)}\right)}^{2} \end{array}$$ (4.7)

Since the second term on the right side of (4.7) is always positive. Hence, $\text{MSE}\left({\hat{\pi }}_{A(crss)}\right)<\text{MSE}\left({\hat{\pi }}_{Y(srs)}\right)$, provided *p* ≠ 0.5. In other words, the expression (4.7) reveals that the proposed estimator ${\hat{\pi }}_{A(crss)}$ is more reliable (having less risk) than ${\hat{\pi }}_{Y(srs)}$.

The relative efficiency of ${\hat{\pi }}_{A(rss)}$ with respect to ${\hat{\pi }}_{Y(srs)}$ can be measured by examining

$$\begin{array}{c} \text{RE}[{\hat{\pi }}_{A(crss)},{\hat{\pi }}_{Y(srs)}]=\frac{\text{MSE}({\hat{\pi }}_{Y(srs)})}{\text{MSE}({\hat{\pi }}_{A(crss)})}={\left\{1-\frac{\lambda^{2}\sum_{i=1}^{m}{\left(\tau_{y[i]}-\tau_{x(i)}\right)}^{2}}{m^{2}{\left(2p-1\right)}^{2}\text{MSE}\left({\hat{\pi }}_{Y(srs)}\right)}\right\}}^{-1} \end{array}$$ (4.8)

The numerical results, for different choices of *m* and *p* when *X* follows (i) normal distribution (ii) uniform distribution, are computed by numerical integration technique using Mathematica Software. The RE results, obtained by using the expression (4.8), are reported in S5-S8 Tables in S1 File. Note that, for the choice *p* = 0.5, RE becomes undefined, so we have omitted RE values against *p* = 0.5. As expected, all results in S5-S8 Tables in S1 File are greater than 1, and RE is an increasing function of *m* i.e., more precise results can be obtained by increasing *m*. It can be observed from S5-S8 Tables in S1 File that there is no symmetry among the RE values, obtained under the interval 0.1 ≤ *p* < 0.5 and 0.5 < *p* ≤ 0.9, as was observed for the case of ${\hat{\pi }}_{\left(crss\right)}$ and ${\hat{\pi }}_{\left(srs\right)}$ (see S1-S4 Tables in S1 File). However, as all RE values are greater than unity, ${\hat{\pi }}_{A(crss)}$ can be considered as an efficient alternative to ${\hat{\pi }}_{Y(srs)}$.

## 5 An application to real data

Following the Reference [24], we have conducted a small scale survey to collect the primary data set of 500 male students in Quaid-i-Azam University, Islamabad. In this survey, each student was asked about his age and a sensitive attribute−whether he has a ‘girl-friend’ or not. On our request, the students spared themselves for this activity and promised to response truthfully via the Warner’s [1] randomizing device with *p* = 0.2. We considered ‘age’ as a concomitant variable *X* and ‘girl-friend’ as a sensitive attribute *Y*. The purpose of this data gathering was to make known of the quantities such as mean and variance of *X* along with proportion of study attribute *Y* and correlation coefficient *ρ*, which are given by $\bar{X}=24$, $\sigma_{x}^{2}=5$, *π* = 0.30 and *ρ* = 0.35 respectively.

Assuming the above population data, we took a concomitant-based ranked set sample of size *k* = 5(2) = 10 as follows: We selected *m*^2^ = 25 students by simple random sample with replacement sampling and randomly partitioned them into 5 sets each of size 5. Furthermore, the students in each set are ranked with respect to *X* and then *i*th ranked student is selected from the *i*th set (*i* = 1, 2, …, 5) to estimate *π*. A layout of CRSS method is given in S9. Table in S1 File, where *Y*_[*i*]*jk*_ denotes *i*th judgment (imperfect) ordered statistic of the student in *j*th set at *k*th cycle and *X*_(*i*)*jk*_ serves *i*th perfect ordered statistic of the student in *j*th set at *k*th cycle. In the final acquired data, we have omitted *j*th set information for the sake of brevity. On the other hand, under simple random sample plan, 6 out of 10 students reported ‘yes’, that is, ${\hat{\lambda }}_{srs}=0.6$. From the data given in S9. Table in S1 File, we have computed some estimates and their associated variances (MSEs) for illustration purpose as given below.

$$\begin{array}{cccc} {\hat{\lambda }}_{\left[crss\right]} & =\frac{1}{10}\sum_{i=1}^{5}\sum_{k=1}^{2}Y_{[i]k}=0.60 & & {\hat{\bar{X}}}_{\left(rss\right)}=\frac{1}{10}\sum_{i=1}^{5}\sum_{k=1}^{2}X_{(i)k}=28.00\\ {\hat{\pi }}_{\left(srs\right)} & =\frac{{\hat{\lambda }}_{srs}-\left(1-p\right)}{2p-1}=0.33 & & {\hat{\pi }}_{\left(crss\right)}=\frac{{\hat{\lambda }}_{rss}-\left(1-p\right)}{2p-1}=0.33\\ {\hat{\lambda }}_{r,rss} & ={\hat{\lambda }}_{\left[rss\right]}(\frac{\bar{X}}{{\hat{\bar{X}}}_{\left(rss\right)}})=0.51 & & {\hat{\pi }}_{A(crss)}=\frac{{\hat{\lambda }}_{r,rss}-\left(1-p\right)}{2p-1}=0.48 \end{array}$$

From (3.1) and (3.6), we have $\hat{\text{Var}}\left({\hat{\pi }}_{\left(srs\right)}\right)=0.07$ and $\hat{\text{Var}}\left({\hat{\pi }}_{\left(crss\right)}\right)=0.06$. Similarly, from (4.2) and (4.7), we have $\hat{\text{MSE}}\left({\hat{\pi }}_{Y(srs)}\right)=0.074$ and $\hat{\text{MSE}}\left({\hat{\pi }}_{Y(crss)}\right)=0.070$.

As expected, both ${\hat{\pi }}_{\left(srs\right)}$ and ${\hat{\pi }}_{\left(crss\right)}$ estimates are very close to true *π*. It can be observed that $\hat{\text{Var}}\left({\hat{\pi }}_{\left(crss\right)}\right)$ is less than $\hat{\text{Var}}\left({\hat{\pi }}_{\left(srs\right)}\right)$. This supports ${\hat{\pi }}_{\left(crss\right)}$ instead of ${\hat{\pi }}_{\left(srs\right)}$ for the estimation of *π*. Similarly, $\hat{\text{MSE}}\left({\hat{\pi }}_{A(crss)}\right)$ is less than $\hat{\text{MSE}}\left({\hat{\pi }}_{Y(srs)}\right)$ indicates that proposed ratio method for estimating sensitive attribute is better than ordinary estimator given in the Reference [22]. Moreover, we can expect further improvement in these results by taking into account multiple-concomitants situation in the present study, as advised in the Reference [14], which is in progress.

## 6 Conclusion

In this study, we have suggested an efficient alternative to Warner’s model [1] for estimating sensitive proportion under CRSS plan. Additionally, a new estimator that based on ratio method has also been proposed using CRSS and compared with its SRS counterpart given in [22]. Both mathematical and numerical results support our proposed estimators.

In future research, it would be interesting to explore effects on the results in Bayesian framework under ranked set sampling methods.

Finally, we would like to discuss the case of generalizing the proposed ratio estimator ${\hat{\lambda }}_{\left[r,rss\right]}$ of λ so as to incorporate concomitant information along with its known parameters for further enhancing accuracy of the results. It is worth-mention that one can also estimate λ via exponential ratio estimator [25]. Thus, two general families of estimators for λ are presented as

$$\begin{array}{c} {\hat{\lambda }}_{\left[r,crss\right]}=\{\begin{array}{c} {\hat{\lambda }}_{\left[rss\right]}\{\frac{a\bar{X}+b}{a{\bar{x}}_{\left(rss\right)}+b}\}\;\text{Ratio}\;\text{family}\\ \text{and}\\ {\hat{\lambda }}_{\left[rss\right]}\exp\{\frac{a(\bar{X}-{\bar{x}}_{\left(rss\right)})}{a(\bar{X}+{\bar{x}}_{\left(rss\right)})+2b}\}\;\text{Exponential}\;\text{family} \end{array} \end{array}$$

Where *a* ≠ 0 and *b* are known parameters of *X*. For specific problem, any one of them can be selected to better estimate sensitive proportion as oppose to existing [25] procedure. Hence, this study has provided different options to the experimenter for obtaining precise measure of sensitive proportion.

## Supporting information

S1 Appendix (PDF)

S1 Fig Probability tree diagram of *i*th response. (TIF)

S1 File Relative efficiencies of the proposed methods and a layout of real data set. (PDF)
