# Supplementary material for: Sensitive proportion in ranked set sampling
Source: PLoS One. 2021 Aug 31;16(8):e0256699. doi: 10.1371/journal.pone.0256699 (PMC8407548; doi:10.1371/journal.pone.0256699)
Supplement: S1 Appendix — (PDF) [file pone.0256699.s001.pdf]

Appendix

We derive the expressions for $\mathrm{E}(\xi_i^2), i = 0, 1$ and $\mathrm{E}(\xi_o\xi_1)$ as left for proof in the Section 4. Let

$$\xi_o = \frac{\hat{\lambda}_{[rss]} - \lambda}{\lambda} \quad \text{and} \quad \xi_1 = \frac{\hat{\bar{X}}_{(rss)} - \bar{X}}{\bar{X}}$$

such that $\mathrm{E}(\xi_0) = 0 = \mathrm{E}(\xi_1)$. From the Reference [1,2] and taking into account RR procedure, we have

$$\begin{aligned}
\mathrm{E}(\xi_o^2) &= \frac{\mathrm{Var}(\hat{\lambda}_{[rss]})}{\lambda^2} = \frac{1}{\lambda^2 m^2}\sum_{i=1}^{m}\mathrm{Var}(\hat{\lambda}_{[i]}) \\
&= \frac{1}{\lambda^2 mk}\sum_{i=1}^{m}\lambda_{[i]}(1-\lambda_{[i]}) \\
&= \frac{1}{\lambda^2 mk}\left\{m\lambda(1-\lambda) - \sum_{i=1}^{m}(\lambda_{[i]}-\lambda)^2\right\} \\
&= \frac{1}{mk\lambda^2}\left\{m((2p-1)^2\pi(1-\pi) + p(1-p)) - (2p-1)^2\sum_{i=1}^{m}d_{y[i]}^2\right\} \\
&= \frac{1}{k}\left\{\frac{1}{\lambda^2}((2p-1)^2\pi(1-\pi) + p(1-p)) - \frac{1}{m}\sum_{i=1}^{m}\tau_{y[i]}^2\right\}
\end{aligned}$$

$$\begin{aligned}
\mathrm{E}(\xi_1^2) &= \frac{1}{\bar{X}^2}\mathrm{Var}(\hat{\bar{X}}_{(rss)}) \\
&= \frac{1}{k\bar{X}^2}\left\{\sigma_x^2 - \frac{1}{m}\sum_{i=1}^{m}d_{x(i)}^2\right\} \\
&= \frac{1}{k}\left\{C_x^2 - \frac{1}{m}\sum_{i=1}^{m}\tau_{x(i)}^2\right\},
\end{aligned}$$

$$\begin{aligned}
\mathrm{E}(\xi_0\xi_1) &= \frac{1}{\lambda\bar{X}}\mathrm{Cov}(\hat{\lambda}_{[rss]}, \hat{\bar{X}}_{(rss)}) \\
&= \frac{(2p-1)}{k\lambda\bar{X}}\left\{\sigma_{yx} - \frac{1}{m}\sum_{i=1}^{m}d_{yx[i]}\right\} \\
&= \frac{1}{k}\left\{\frac{\pi(2p-1)}{\lambda}C_{xy} - \frac{1}{m}\sum_{i=1}^{m}\tau_{xy[i]}\right\},
\end{aligned}$$

where $\tau_{x(i)} = d_{x(i)}/\bar{X}, \qquad \tau_{y[i]} = (2p-1)d_{y[i]}/\lambda, \qquad \tau_{yx[i]} = (2p-1)d_{xy[i]}/(\lambda\bar{X})$

and σ_{xy} is covariance between X and Y, whereas the quantities C_x and C_{xy} are coefficient of variation as earlier defined.

References

1. **Dell TR and Clutter JL (1972)**. Ranked set sampling theory with order statistics background. Biometrics 545-555.

2. **Samawi HM and Muttlak HA (1996)**. Estimation of ratio using rank set sampling. Biometrical Journal 38: 753-764.
