# Supplementary figures and images for: Sensitive proportion in ranked set sampling
Source: PLoS One. 2021 Aug 31;16(8):e0256699. doi: 10.1371/journal.pone.0256699 (PMC8407548; doi:10.1371/journal.pone.0256699)

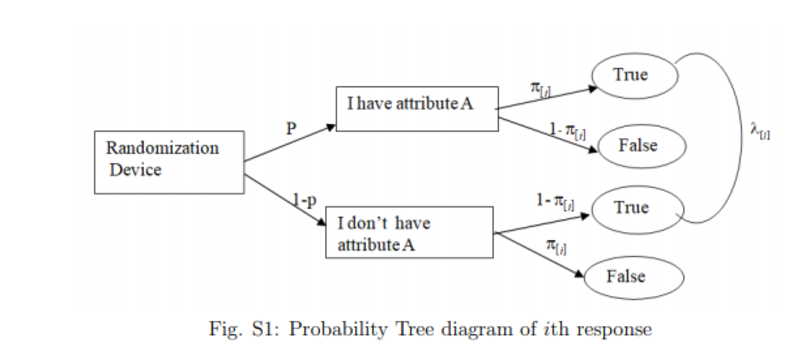

Supplement: S1 Fig — (TIF) [file pone.0256699.s002.tif]
