# Supplementary material for: Sensitive proportion in ranked set sampling
Source: PLoS One. 2021 Aug 31;16(8):e0256699. doi: 10.1371/journal.pone.0256699 (PMC8407548; doi:10.1371/journal.pone.0256699)
Supplement: S1 File — (PDF) [file pone.0256699.s003.pdf]

Table S1 : RE$[\hat{\pi}_{(crss)}, \hat{\pi}_{(srs)}]$ for different β_0, β_1 when $0.1 \leq p \leq 0.9$ and $m = 2$

Distribution	Link	β_0	β_1	ρ	π	$p = 0.1$	$p = 0.2$	$p = 0.3$	$p = 0.4$	$p = 0.5$	$p = 0.6$	$p = 0.7$	$p = 0.8$	$p = 0.9$
Normal	Logit	−10	7	0.731	0.710	1.128	1.064	1.026	1.006	1.000	1.006	1.026	1.064	1.128
		−5	5	0.636	0.826	1.068	1.032	1.013	1.003	1.000	1.003	1.013	1.032	1.068
		−2	6	0.467	0.944	1.013	1.005	1.002	1.000	1.000	1.000	1.002	1.005	1.013
	Probit	−10	7	0.730	0.709	1.128	1.064	1.026	1.006	1.000	1.006	1.026	1.064	1.128
		−5	5	0.636	0.826	1.068	1.032	1.013	1.003	1.000	1.003	1.013	1.032	1.068
		−2	6	0.468	0.944	1.013	1.005	1.002	1.000	1.000	1.000	1.002	1.005	1.013
Uniform	Logit	−10	7	0.103	0.007	1.000	1.000	1.000	1.000	1.000	1.000	1.000	1.000	1.000
		−5	5	0.376	0.137	1.022	1.010	1.004	1.001	1.000	1.001	1.004	1.010	1.022
		−2	6	0.592	0.648	1.073	1.039	1.017	1.004	1.000	1.004	1.017	1.039	1.073
	Probit	−10	7	0.102	0.005	1.000	1.000	1.000	1.000	1.000	1.000	1.000	1.000	1.000
		−5	5	0.391	0.144	1.024	1.011	1.004	1.001	1.000	1.001	1.004	1.011	1.024
		−2	6	0.575	0.647	1.072	1.037	1.016	1.004	1.000	1.004	1.016	1.037	1.072

Table S2 : RE$[\hat{\pi}_{(crss)}, \hat{\pi}_{(srs)}]$ for different β_0, β_1 when $0.1 \leq p \leq 0.9$ and $m = 3$

Distribution	Link	β_0	β_1	ρ	π	$p = 0.1$	$p = 0.2$	$p = 0.3$	$p = 0.4$	$p = 0.5$	$p = 0.6$	$p = 0.7$	$p = 0.8$	$p = 0.9$
Normal	Logit	−10	7	0.731	0.710	1.218	1.105	1.043	1.010	1.000	1.010	1.043	1.105	1.218
		−5	5	0.636	0.826	1.120	1.055	1.021	1.005	1.000	1.005	1.021	1.055	1.120
		−2	6	0.467	0.944	1.025	1.010	1.003	1.001	1.000	1.001	1.003	1.010	1.025
	Probit	−10	7	0.730	0.709	1.218	1.105	1.043	1.010	1.000	1.010	1.043	1.105	1.218
		−5	5	0.636	0.826	1.120	1.055	1.021	1.005	1.000	1.005	1.021	1.055	1.120
		−2	6	0.468	0.944	1.025	1.010	1.003	1.001	1.000	1.001	1.003	1.010	1.025
Uniform	Logit	−10	7	0.103	0.007	1.000	1.000	1.000	1.000	1.000	1.000	1.000	1.000	1.000
		−5	5	0.376	0.137	1.034	1.015	1.006	1.001	1.000	1.001	1.006	1.015	1.034
		−2	6	0.592	0.648	1.123	1.064	1.027	1.006	1.000	1.006	1.027	1.064	1.123
	Probit	−10	7	0.102	0.005	1.000	1.000	1.000	1.000	1.000	1.000	1.000	1.000	1.000
		−5	5	0.391	0.144	1.038	1.017	1.007	1.002	1.000	1.002	1.007	1.017	1.038
		−2	6	0.575	0.647	1.115	1.060	1.025	1.006	1.000	1.006	1.025	1.060	1.115

Table S3 : RE$[\hat{\pi}_{(crss)}, \hat{\pi}_{(srs)}]$ for different β_0, β_1 when $0.1 \leq p \leq 0.9$ and $m = 4$

Distribution	Link	β_0	β_1	ρ	π	$p = 0.1$	$p = 0.2$	$p = 0.3$	$p = 0.4$	$p = 0.5$	$p = 0.6$	$p = 0.7$	$p = 0.8$	$p = 0.9$
Normal	Logit	−10	7	0.731	0.710	1.286	1.134	1.053	1.012	1.000	1.012	1.053	1.134	1.286
		−5	5	0.636	0.826	1.161	1.072	1.028	1.006	1.000	1.006	1.028	1.072	1.161
		−2	6	0.467	0.944	1.036	1.014	1.005	1.001	1.000	1.001	1.005	1.014	1.036
	Probit	−10	7	0.730	0.709	1.286	1.134	1.053	1.012	1.000	1.012	1.053	1.134	1.286
		−5	5	0.636	0.826	1.161	1.072	1.028	1.006	1.000	1.006	1.028	1.072	1.161
		−2	6	0.468	0.944	1.036	1.014	1.005	1.001	1.000	1.001	1.005	1.014	1.036
Uniform	Logit	−10	7	0.103	0.007	1.000	1.000	1.000	1.000	1.000	1.000	1.000	1.000	1.000
		−5	5	0.376	0.137	1.043	1.020	1.007	1.002	1.000	1.002	1.007	1.020	1.043
		−2	6	0.592	0.648	1.152	1.078	1.033	1.008	1.000	1.008	1.033	1.078	1.152
	Probit	−10	7	0.102	0.005	1.000	1.000	1.000	1.000	1.000	1.000	1.000	1.000	1.000
		−5	5	0.391	0.144	1.047	1.021	1.008	1.002	1.000	1.002	1.008	1.021	1.047
		−2	6	0.575	0.647	1.142	1.073	1.031	1.007	1.000	1.007	1.031	1.073	1.142

Table S4 : RE$[\hat{\pi}_{(crss)}, \hat{\pi}_{(srs)}]$ for different β_0, β_1 when $0.1 \leq p \leq 0.9$ and $m = 5$

Distribution	Link	β_0	β_1	ρ	π	$p = 0.1$	$p = 0.2$	$p = 0.3$	$p = 0.4$	$p = 0.5$	$p = 0.6$	$p = 0.7$	$p = 0.8$	$p = 0.9$
Normal	Logit	−10	7	0.731	0.710	1.338	1.156	1.061	1.014	1.000	1.014	1.061	1.156	1.338
		−5	5	0.636	0.826	1.193	1.085	1.033	1.007	1.000	1.007	1.033	1.085	1.193
		−2	6	0.467	0.944	1.058	1.022	1.008	1.002	1.000	1.002	1.008	1.022	1.058
	Probit	−10	7	0.730	0.709	1.338	1.156	1.061	1.014	1.000	1.014	1.061	1.156	1.338
		−5	5	0.636	0.826	1.193	1.085	1.033	1.007	1.000	1.007	1.033	1.085	1.193
		−2	6	0.468	0.944	1.058	1.022	1.008	1.002	1.000	1.002	1.008	1.022	1.058
Uniform	Logit	−10	7	0.103	0.007	1.000	1.000	1.000	1.000	1.000	1.000	1.000	1.000	1.000
		−5	5	0.376	0.137	1.048	1.022	1.008	1.002	1.000	1.002	1.008	1.022	1.048
		−2	6	0.592	0.648	1.173	1.088	1.036	1.009	1.000	1.009	1.036	1.088	1.173
	Probit	−10	7	0.102	0.005	1.000	1.000	1.000	1.000	1.000	1.000	1.000	1.000	1.000
		−5	5	0.391	0.144	1.053	1.024	1.009	1.002	1.000	1.002	1.009	1.024	1.053
		−2	6	0.575	0.647	1.160	1.082	1.034	1.008	1.000	1.008	1.034	1.082	1.160

Table S5 : RE$[\hat{\pi}_{A(crss)}, \hat{\pi}_{Y(srs)}]$ for different β_0, β_1 when $0.1 \leq p \leq 0.9$ and $m = 2$

Distribution	Link	β_0	β_1	ρ	π	$p = 0.1$	$p = 0.2$	$p = 0.3$	$p = 0.4$	$p = 0.5$	$p = 0.6$	$p = 0.7$	$p = 0.8$	$p = 0.9$
Normal	Logit	−10	7	0.731	0.710	1.233	1.172	1.128	1.094	–	1.047	1.030	1.016	1.006
		−5	5	0.636	0.826	1.145	1.111	1.090	1.077	–	1.062	1.059	1.060	1.067
		−2	6	0.467	0.944	1.049	1.047	1.050	1.057	–	1.083	1.106	1.142	1.208
	Probit	−10	7	0.730	0.709	1.233	1.172	1.128	1.094	–	1.047	1.030	1.016	1.006
		−5	5	0.636	0.826	1.146	1.111	1.090	1.077	–	1.062	1.059	1.060	1.067
		−2	6	0.468	0.944	1.050	1.047	1.050	1.057	–	1.083	1.106	1.142	1.208
Uniform	Logit	−10	7	0.103	0.007	1.329	1.235	1.171	1.125	–	1.064	1.042	1.025	1.010
		−5	5	0.376	0.137	1.287	1.225	1.173	1.129	–	1.058	1.032	1.012	1.000
		−2	6	0.592	0.648	1.214	1.174	1.141	1.114	–	1.071	1.054	1.039	1.027
	Probit	−10	7	0.102	0.005	1.330	1.235	1.171	1.125	–	1.064	1.042	1.025	1.010
		−5	5	0.391	0.144	1.286	1.225	1.173	1.129	–	1.058	1.031	1.011	1.000
		−2	6	0.575	0.647	1.203	1.167	1.137	1.112	–	1.073	1.057	1.043	1.031

Table S6 : RE$[\hat{\pi}_{A(crss)}, \hat{\pi}_{Y(srs)}]$ for different β_0, β_1 when $0.1 \leq p \leq 0.9$ and $m = 3$

Distribution	Link	β_0	β_1	ρ	π	$p = 0.1$	$p = 0.2$	$p = 0.3$	$p = 0.4$	$p = 0.5$	$p = 0.6$	$p = 0.7$	$p = 0.8$	$p = 0.9$
Normal	Logit	−10	7	0.731	0.710	1.407	1.288	1.207	1.149	–	1.072	1.047	1.030	1.021
		−5	5	0.636	0.826	1.248	1.182	1.144	1.120	–	1.097	1.095	1.099	1.118
		−2	6	0.467	0.944	1.081	1.074	1.078	1.088	–	1.130	1.168	1.232	1.353
	Probit	−10	7	0.730	0.709	1.407	1.287	1.207	1.148	–	1.072	1.047	1.030	1.021
		−5	5	0.636	0.826	1.248	1.182	1.144	1.120	–	1.097	1.095	1.099	1.118
		−2	6	0.468	0.944	1.081	1.074	1.078	1.088	–	1.130	1.168	1.232	1.353
Uniform	Logit	−10	7	0.103	0.007	1.564	1.385	1.273	1.198	–	1.101	1.068	1.042	1.020
		−5	5	0.376	0.137	1.287	1.242	1.203	1.170	–	1.120	1.104	1.095	1.101
		−2	6	0.592	0.648	1.159	1.134	1.123	1.125	–	1.182	1.256	1.400	1.732
	Probit	−10	7	0.102	0.005	1.569	1.387	1.274	1.198	–	1.102	1.069	1.043	1.020
		−5	5	0.391	0.144	1.281	1.238	1.201	1.169	–	1.121	1.106	1.099	1.107
		−2	6	0.575	0.647	1.153	1.131	1.121	1.124	–	1.181	1.253	1.390	1.692

Table S7 : RE$[\hat{\pi}_{A(crss)}, \hat{\pi}_{Y(srs)}]$ for different β_0, β_1 when $0.1 \leq p \leq 0.9$ and $m = 4$

Distribution	Link	β_0	β_1	ρ	π	$p = 0.1$	$p = 0.2$	$p = 0.3$	$p = 0.4$	$p = 0.5$	$p = 0.6$	$p = 0.7$	$p = 0.8$	$p = 0.9$
Normal	Logit	−10	7	0.731	0.710	1.542	1.371	1.261	1.185	–	1.089	1.059	1.041	1.037
		−5	5	0.636	0.826	1.327	1.232	1.180	1.148	–	1.119	1.118	1.127	1.158
		−2	6	0.467	0.944	1.104	1.092	1.096	1.109	–	1.161	1.210	1.293	1.461
	Probit	−10	7	0.730	0.709	1.542	1.371	1.261	1.185	–	1.089	1.059	1.041	1.037
		−5	5	0.636	0.826	1.327	1.232	1.180	1.148	–	1.119	1.118	1.127	1.158
		−2	6	0.468	0.944	1.104	1.092	1.096	1.109	–	1.161	1.210	1.293	1.461
Uniform	Logit	−10	7	0.103	0.007	1.851	1.529	1.266	1.191	–	1.095	1.056	1.045	1.018
		−5	5	0.376	0.137	1.674	1.467	1.260	1.154	–	1.087	1.047	1.028	1.003
		−2	6	0.592	0.648	1.433	1.266	1.214	1.171	–	1.107	1.082	1.062	1.044
	Probit	−10	7	0.102	0.005	1.851	1.529	1.266	1.191	–	1.095	1.056	1.045	1.018
		−5	5	0.391	0.144	1.673	1.464	1.260	1.154	–	1.087	1.047	1.027	1.002
		−2	6	0.575	0.647	1.433	1.260	1.210	1.169	–	1.108	1.082	1.064	1.047

Table S8 : RE$[\hat{\pi}_{A(crss)}, \hat{\pi}_{Y(srs)}]$ for different β_0, β_1 when $0.1 \leq p \leq 0.9$ and $m = 5$

Distribution	Link	β_0	β_1	ρ	π	$p = 0.1$	$p = 0.2$	$p = 0.3$	$p = 0.4$	$p = 0.5$	$p = 0.6$	$p = 0.7$	$p = 0.8$	$p = 0.9$
Normal	Logit	−10	7	0.731	0.710	1.652	1.435	1.301	1.210	–	1.101	1.062	1.051	1.052
		−5	5	0.636	0.826	1.390	1.271	1.207	1.168	–	1.135	1.134	1.147	1.188
		−2	6	0.467	0.944	1.124	1.106	1.109	1.123	–	1.183	1.239	1.338	1.544
	Probit	−10	7	0.730	0.710	1.653	1.435	1.301	1.210	–	1.101	1.069	1.051	1.052
		−5	5	0.636	0.826	1.390	1.271	1.207	1.168	–	1.135	1.134	1.147	1.188
		−2	6	0.468	0.944	1.124	1.107	1.109	1.123	–	1.183	1.239	1.338	1.544
Uniform	Logit	−10	7	0.103	0.007	1.983	1.614	1.413	1.286	–	1.137	1.088	1.051	1.020
		−5	5	0.376	0.137	1.808	1.583	1.419	1.296	–	1.125	1.067	1.026	1.005
		−2	6	0.592	0.648	1.533	1.413	1.324	1.255	–	1.155	1.119	1.088	1.063
	Probit	−10	7	0.102	0.005	1.988	1.615	1.413	1.286	–	1.137	1.088	1.051	1.021
		−5	5	0.391	0.144	1.807	1.585	1.421	1.297	–	1.124	1.066	1.024	1.004
		−2	6	0.575	0.647	1.515	1.403	1.318	1.252	–	1.157	1.121	1.92	1.066

Table S9 : A layout of CRSS for $m = 5$ and $n = 2$

Cycle(n)	Set(m)	Set Units					Acquired data
1	1	$\boxed{(Y_{[1]11}, X_{(1)11})}$	$(Y_{[2]11}, X_{(2)11})$	$(Y_{[3]11}, X_{(3)11})$	$(Y_{[4]11}, X_{(4)11})$	$(Y_{[5]11}, X_{(5)11})$	$Y_{[1]1} = 1, X_{(1)1} = 28$
	2	$(Y_{[1]21}, X_{(1)21})$	$\boxed{(Y_{[2]21}, X_{(2)21})}$	$(Y_{[3]21}, X_{(3)21})$	$(Y_{[4]21}, X_{(4)21})$	$(Y_{[5]21}, X_{(5)21})$	$Y_{[2]1} = 0, X_{(2)1} = 24$
	3	$(Y_{[1]31}, X_{(1)31})$	$(Y_{[2]31}, X_{(2)31})$	$\boxed{(Y_{[3]31}, X_{(3)31})}$	$(Y_{[4]31}, X_{(4)31})$	$(Y_{[5]31}, X_{(5)31})$	$Y_{[3]1} = 1, X_{(3)1} = 26$
	4	$(Y_{[1]41}, X_{(1)41})$	$(Y_{[2]41}, X_{(2)41})$	$(Y_{[3]41}, X_{(3)41})$	$\boxed{(Y_{[4]41}, X_{(4)41})}$	$(Y_{[5]41}, X_{(5)41})$	$Y_{[4]1} = 0, X_{(4)1} = 29$
	5	$(Y_{[1]51}, X_{(1)51})$	$(Y_{[2]51}, X_{(2)51})$	$(Y_{[3]51}, X_{(3)51})$	$(Y_{[4]51}, X_{(4)51})$	$\boxed{(Y_{[5]51}, X_{(5)51})}$	$Y_{[5]1} = 0, X_{(5)1} = 29$
2	1	$\boxed{(Y_{[1]12}, X_{(1)12})}$	$(Y_{[2]12}, X_{(2)12})$	$(Y_{[3]12}, X_{(3)12})$	$(Y_{[4]12}, X_{(4)12})$	$(Y_{[5]12}, X_{(5)12})$	$Y_{[1]2} = 0, X_{(1)2} = 31$
	2	$(Y_{[1]22}, X_{(1)22})$	$\boxed{(Y_{[2]22}, X_{(2)22})}$	$(Y_{[3]22}, X_{(3)22})$	$(Y_{[4]22}, X_{(4)22})$	$(Y_{[5]22}, X_{(5)22})$	$Y_{[2]2} = 1, X_{(2)2} = 30$
	3	$(Y_{[1]32}, X_{(1)32})$	$(Y_{[2]32}, X_{(2)32})$	$\boxed{(Y_{[3]22}, X_{(3)22})}$	$(Y_{[4]32}, X_{(4)32})$	$(Y_{[5]32}, X_{(5)32})$	$Y_{[3]2} = 1, X_{(3)2} = 29$
	4	$(Y_{[1]42}, X_{(1)42})$	$(Y_{[2]42}, X_{(2)42})$	$(Y_{[3]42}, X_{(3)42})$	$\boxed{(Y_{[4]42}, X_{(4)42})}$	$(Y_{[5]42}, X_{(5)42})$	$Y_{[4]2} = 1, X_{(4)2} = 24$
	5	$(Y_{[1]52}, X_{(1)52})$	$(Y_{[2]52}, X_{(2)52})$	$(Y_{[3]52}, X_{(3)52})$	$(Y_{[4]52}, X_{(4)52})$	$\boxed{(Y_{[5]52}, X_{(5)52})}$	$Y_{[5]2} = 1, X_{(5)2} = 29$
